# Case Report: Self-Resolved Macular Edema Secondary to Congenital Retinal Macrovessels

**DOI:** 10.3389/fmed.2021.771007

**Published:** 2022-01-17

**Authors:** Fang Zheng, Kairan Lai, Houfa Yin, Jingliang He, Yufeng Xu, Panpan Ye

**Affiliations:** Eye Center, The Second Affiliated Hospital Zhejiang University School of Medicine, Zhejiang University, Hangzhou, China

**Keywords:** congenital retinal macrovessels, macular edema, OCT, OCTA, FFA

## Abstract

**Purpose:**

To report a case of macular edema secondary to congenital retinal macrovessels (CRMs), which resolved spontaneously without any treatment.

**Observations:**

A 39-year-old female presented with blurry vision of the right eye for one day. Fundus examination revealed a branch of artery and vein of the inferior retinal arcade crossing the horizontal raphe. Optical coherence tomography (OCT) through the fovea showed cystoid macular edema in the outer plexiform layer. However, no leakage of the vessels was noticed by fundus fluorescein angiography (FFA). Observation was recommended with close follow-up. Two weeks later, the patient returned with good visual acuity, and the macular edema was resolved spontaneously.

**Conclusions:**

Macular edema is a possible complication of CRM by increasing retinal capillary hydrostatic pressure. Treatment is not necessary for this kind of macular edema if no leakage of the vessels is noticed on FFA.

## Introduction

Congenital retinal macrovessels (CRMs) are usually unilateral, aberrant, large branches of retinal vessels crossing the horizontal raphe (1). These could be arteries or veins, but commonly veins are involved. CRM is rare and typically asymptomatic. CRM may be incidentally complicated with serous macular detachment (2), hemorrhage (3), macroaneurysms (4), exudation, or foveal cyst (1). In this article, we report a case of spontaneously resolved macular edema caused by CRMs, comprising an artery and a vein.

## Case Report

**Figure 1:**
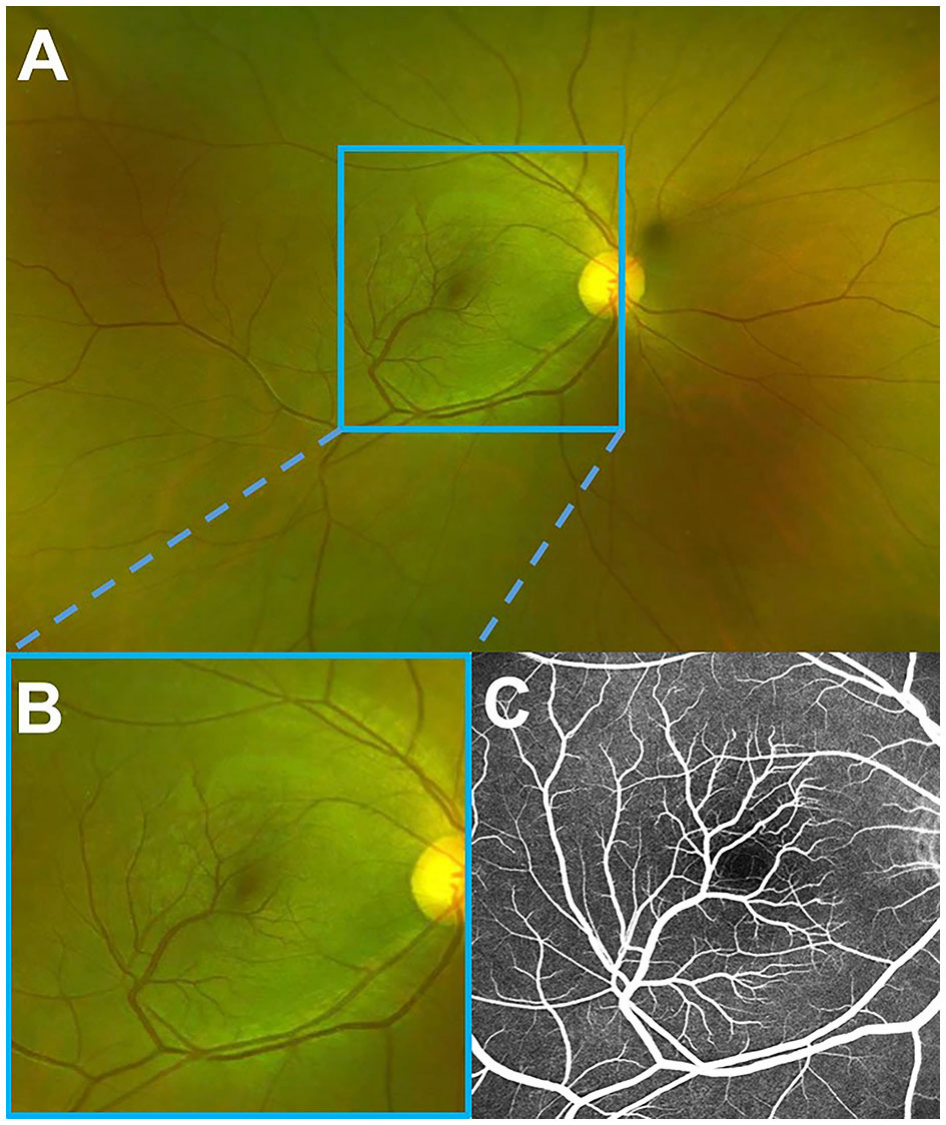
A fundus photo and fluorescein angiography of the right eye. **(A)** Optos wide-field fundus image showing a branch of each artery and vein of the inferior retinal arcade, extending into macula with a sharp angle. **(B)** Magnified image demonstrating a better view of the macula. **(C)** Late fluorescein angiography, revealing no leakage of these vessels, the formation of the fovea avascular zone by the terminal arterioles, and the fovea-spared routine of the vein.

**Figure 2:**
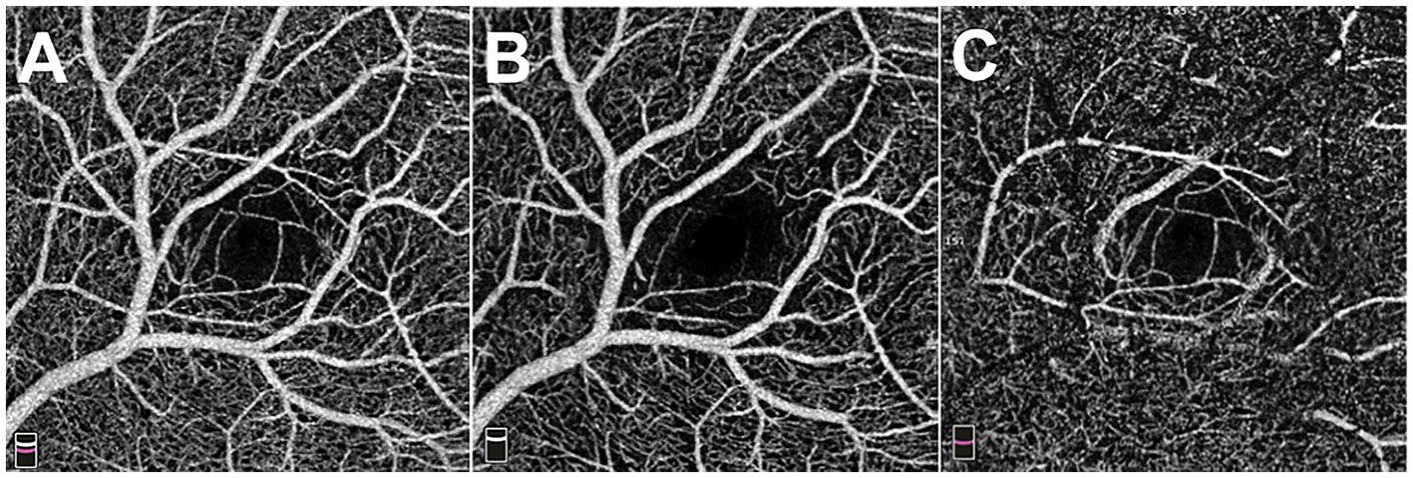
The 3 x 3 mm optical coherence tomography angiography images of the macula. **(A)** Total retinal flow of macula. **(B)** Superficial retinal flow of macula. **(C)** Deep retinal flow of macula.

**Figure 3:**
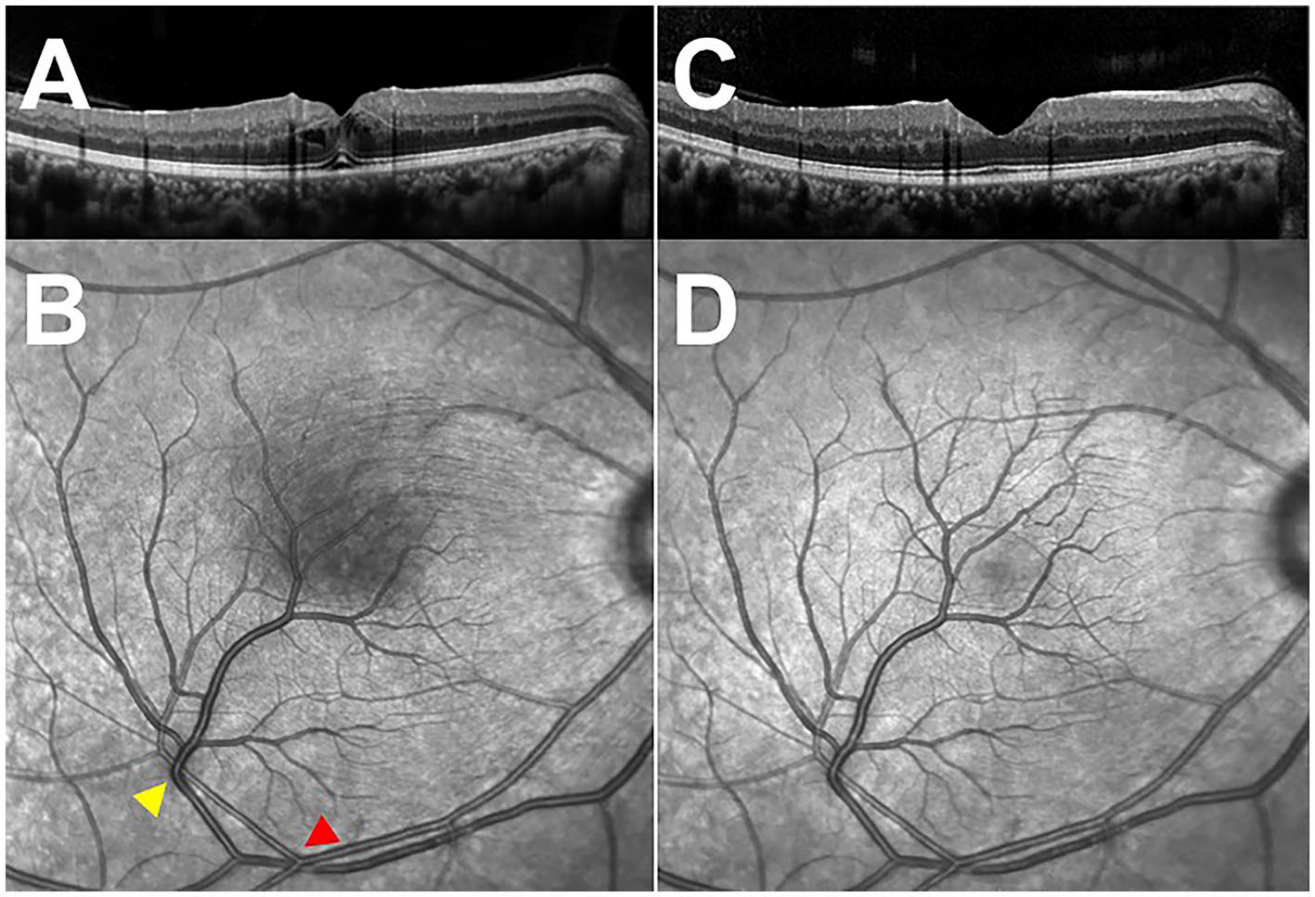
Optical coherence tomography (OCT) through the fovea and infra-red images of the macula. **(A)** Initial OCT of the right eye, demonstrating cystoid macular edema. **(B)** Infra-red image of the macula, showing the sites of arteriovenous impression (arrowheads) and the shadow of the swollen never-fiber layer on retinal pigment epithelium (RPE). **(C)** OCT of the right eye at 2-week follow-up visit, revealing the absorption of macular edema. **(D)** Infra-red image of the macula at a 2-week follow-up visit, exhibiting the disappearance of the shadow.

A 39-year-old female presented with blurry vision of the right eye for one day. The best corrected visual acuity (BCVA) of the right eye was 20/25. She had no history of hypertension or diabetes, or any other systemic disease. The anterior segment in both eyes revealed no abnormal findings. Fundus examination revealed a branch of artery and vein of the inferior retinal arcade extending into macula with a sharp angle (Figures 1A,B) (Optos, Optos PLC, Dunfermline, UK). Fundus fluorescein angiography (FFA) revealed no arteriovenous anastomoses and no leakage of these vessels, the formation of fovea avascular zone (FAZ) by the terminal arterioles, and the fovea-spared routine of the vein (Figure 1C). Macular 3 mm x 3 mm optical coherence tomography (OCT) angiography (AngioVue, Optovue RTVue XR 100; AVANTI, Inc) confirmed the preservation of FAZ with more vascular details (Figure 2A). The vein remains in the superficial layer of the retina (Figure 2B), and the artery strays into the deep capillary plexus (Figure 2C). An infra-red image demonstrated a better view of the entanglement of these two vessels as the vein crossing the artery from below (a red arrowhead) to above (a yellow arrowhead) (Figure 3B). OCT through the fovea showed macular edema (Figure 3A) (Spectralis OCT, Heidelberg Engineering, Inc., Heidelberg, Germany), which may lead to decreased visual acuity. Since there was no leakage of the CRM, no treatment was performed at the initial visit, and a clinical appointment within 2 weeks was scheduled. At the 2-week follow-up visit, the BCVA of the patient improved to 20/20, and macular edema was completely absorbed (Figures 3C,D).

## Discussions

The prevalence of CRMs is approximately 1/200,000 (5). CRM rarely causes vision problems and is usually diagnosed during the routine retinal examination. Vision loss is only encountered when there is an abnormality of the macula. However, the retinal complications are typically incidental concomitant with CRMs, such as age-related macular degeneration, central serous chorioretinopathy, or acute macular neuroretinopathy, according to the previous reports (3). In 1982, Brown et al. reported seven patients with CRM, two of them had foveolar cysts based on fundus examination (1). Unlike our patient, the foveolar cyst in Brown's paper remained unchanged during the follow-up visit. According to our best knowledge, this is the first CRM case presenting with macular edema so far.

Macular edema can be simply defined as an excess of fluid within the retinal interstitial space. The main mechanisms for keeping the retina in a dehydrated state are the presence of inner and outer blood-retinal barriers (BRBs) and the one-way movement of fluid across the retinal pigment epithelium (RPE) (6). The inner barrier is made of an endothelial tight junction of the retinal vessels; the outer barrier is produced by the tight connection of RPE cells. Since there was no abnormality of RPE in this patient, the possible factors causing macular edema include abnormality in BRB permeability, capillary hydrostatic pressure, tissue hydrostatic pressure, tissue osmotic pressure, and plasma osmotic pressure (7). This patient had no systemic disease that might influence inner BRB permeability. The FFA finding also confirmed intact endothelial tight junction of retinal vessels by showing no leakage. Tissue hydrostatic pressure and osmotic pressure are unlikely to change when no inflammation is involved. The most reasonable explanation is increased capillary hydrostatic pressure, given the abnormal vascular course of CRM in this patient. Especially with the presence of the arteriovenous impression, the hydrostatic pressure is more vulnerable to a subtle change of systemic blood pressure. Erol et al. described a case of spontaneous regression of macular edema secondary to arteriovenous malformation (AVM) (8). Like in our case, the macular edema regressed without any treatment in 3 days. The authors suggested increase hydrostatic pressure in the abnormal capillaries might be the cause of macular edema. With compensation of healthy RPE-pumping fluids into the retina and back to normal hydrostatic pressure, the macular edema resolved spontaneously.

Increased vascular endothelial growth factor increases vascular permeability and promotes angiogenesis (9). Intravitreal anti-VEGF therapy is now considered the gold standard for treating macular edema caused by various retinal disorders. The fundamental principle of anti-VEGF treatment is to decrease the VEGF level of the eye to prevent leakage caused by increased BRB permeability like in diabetic retinopathy and retinal vein occlusion or inhibition of neovascularization like in wet age-related macular degeneration. When there is no evidence of increased BRB permeability or neovascularization, anti-VEGF is unnecessary even when macular edema is present, like in non-proliferative Type 2 macular telangiectasia, retinitis pigmentosa, and vitelliform macular degeneration (10). In our patient, FFA did not reveal any leakage of vessels, suggesting intact endothelial junction of capillaries, which do not require anti-VEGF treatment. Complete resolution of macular edema and vision recovery further support this perspective.

In conclusion, we described the clinical features of a rare CRM case with macular edema. The hydrostatic pressure change in these vessels is thought to cause macular edema. No anti-VEGF treatment is needed, and the macula edema can resolve spontaneously. However, long-term self-monitoring is recommended since the anatomic abnormality is permanent.

## Data Availability Statement

The original contributions presented in the study are included in the article/supplementary material, further inquiries can be directed to the corresponding author.

## Ethics Statement

The studies involving human participants were reviewed and approved by The Second Affiliated Hospital of Zhejiang University School of Medicine Review Board and Ethics Committee. Written informed consent for participation was not required for this study in accordance with the national legislation and the institutional requirements.

## Author Contributions

All authors listed have made a substantial, direct, and intellectual contribution to the work and approved it for publication.

## Funding

Research was supported by Zhejiang Natural Science Foundation Project of China (No. LY18H120001), National Natural Youth Science Foundation Project of China (No. 31500795), and Zhejiang University Foundation Project (No. 2020-518052-0054).

## Conflict of Interest

The authors declare that the research was conducted in the absence of any commercial or financial relationships that could be construed as a potential conflict of interest.

## Publisher's Note

All claims expressed in this article are solely those of the authors and do not necessarily represent those of their affiliated organizations, or those of the publisher, the editors and the reviewers. Any product that may be evaluated in this article, or claim that may be made by its manufacturer, is not guaranteed or endorsed by the publisher.

## References

[1] Brown GC Donoso LA Magargal LE Goldberg RE Sarin LK. Congenital retinal macrovessels. Arch Ophthalmol. (1982) 100:1430–6. 10.1001/archopht.1982.01030040408006 7115168

[2] Arai J Kasuga Y Koketsu M Yoshimura N. Development and spontaneous resolution of serous retinal detachment in a patient with a congenital retinal macrovessel. Retina. (2000) 20:674–6. 10.1097/00006982-200006000-00018 11131426

[3] Pichi F Freund KB Ciardella A Morara M Abboud EB Ghazi N. Congenital Retinal Macrovessel and the Association of Retinal Venous Malformations With Venous Malformations of the Brain. JAMA Ophthalmol. (2018) 136:372–9. 10.1001/jamaophthalmol.2018.0150 29494725 PMC5876911

[4] Sebrow DB Cunha de Souza E Belúcio Neto J Roizenblatt M Zett Lobos C Paulo Bonomo P. Macroaneurysms associated with congenital retinal macrovessels. Retin Cases Brief Rep. (2020) 14:61–5. 10.1097/ICB.0000000000000619 28799971 PMC5807243

[5] Petropoulos IK Petkou D Theoulakis PE Kordelou A Pournaras CJ Katsimpris JM. Congenital retinal macrovessels: description of three cases and review of the literature. Klin Monbl Augenheilkd. (2008) 225:469–72. 10.1055/s-2008-1027265 18454401

[6] Marmor MF. Mechanisms of fluid accumulation in retinal edema. Doc Ophthalmol. (1999) 97:239–49. 10.1023/A:1002192829817 10896337

[7] Cunha-Vaz J. Mechanisms of retinal fluid accumulation and blood-retinal barrier breakdown. Dev Ophthalmol. (2017) 58:11–20. 10.1159/000455265 28351041

[8] Erol MK Dogan B Çoban DT Sayran H Özdemir O. Spontaneous regression of macular edema secondary to arteriovenous malformation. Retina-Vitreus. (2015) 217–20.

[9] Melincovici CS Bosca AB Susman S. Vascular endothelial growth factor (VEGF) - key factor in normal and pathological angiogenesis. Rom J Morphol Embryol. (2018) 59:455–67. 30173249

[10] Charbel Issa P Kupitz EH Heeren TF Holz FG. Treatment for macular telangiectasia type 2. Dev Ophthalmol. (2016) 55:189–95. 10.1159/000431263 26501828

